# Mobile Functional Reach Test in People Who Suffer Stroke: A Pilot Study

**DOI:** 10.2196/rehab.4102

**Published:** 2015-06-11

**Authors:** Jose Antonio Merchán-Baeza, Manuel González-Sánchez, Antonio Cuesta-Vargas

**Affiliations:** ^1^Departamento de Fisioterapia, Facultad de Ciencias de la Salud, Andalucia Tech, Cátedra de Fisioterapia y Discapacidad, Instituto de Biomedicina de Málaga (IBIMA), Grupo de Clinimetria (FE-14)Universidad de MálagaMálagaSpain; ^2^School of Clinical Sciences of the Faculty of HealthQueensland University of TechnologyBrisbaneAustralia

**Keywords:** mobile health, reliability and validity, elderly, stroke, postural balance

## Abstract

**Background:**

Postural instability is one of the major complications found in people who survive a stroke. Parameterizing the Functional Reach Test (FRT) could be useful in clinical practice and basic research, as this test is a clinically accepted tool (for its simplicity, reliability, economy, and portability) to measure the semistatic balance of a subject.

**Objective:**

The aim of this study is to analyze the reliability in the FRT parameterization using inertial sensor within mobile phones (mobile sensors) for recording kinematic variables in patients who have suffered a stroke. Our hypothesis is that the sensors in mobile phones will be reliable instruments for kinematic study of the FRT.

**Methods:**

This is a cross-sectional study of 7 subjects over 65 years of age who suffered a stroke. During the execution of FRT, the subjects carried two mobile phones: one placed in the lumbar region and the other one on the trunk. After analyzing the data obtained in the kinematic registration by the mobile sensors, a number of direct and indirect variables were obtained. The variables extracted directly from FRT through the mobile sensors were distance, maximum angular lumbosacral/thoracic displacement, time for maximum angular lumbosacral/thoracic displacement, time of return to the initial position, and total time. Using these data, we calculated speed and acceleration of each. A descriptive analysis of all kinematic outcomes recorded by the two mobile sensors (trunk and lumbar) was developed and the average range achieved in the FRT. Reliability measures were calculated by analyzing the internal consistency of the measures with 95% confidence interval of each outcome variable. We calculated the reliability of mobile sensors in the measurement of the kinematic variables during the execution of the FRT.

**Results:**

The values in the FRT obtained in this study (2.49 cm, SD 13.15) are similar to those found in other studies with this population and with the same age range. Intrasubject reliability values observed in the use of mobile phones are all located above 0.831, ranging from 0.831 (time B_C trunk area) and 0.894 (displacement A_B trunk area). Likewise, the observed intersubject values range from 0.835 (time B_C trunk area) and 0.882 (displacement A_C trunk area). On the other hand, the reliability of the FRT was 0.989 (0.981-0.996) and 0.978 (0.970-0.985), intrasubject and intersubject respectively.

**Conclusions:**

We found that mobile sensors in mobile phones could be reliable tools in the parameterization of the Functional Reach Test in people who have had a stroke.

## Introduction

Stroke is the leading cause of severe long-term disability worldwide, and it commonly occurs in people aged 65 years and over [1,2]. Neurological deficits caused by stroke lead to motor, sensory, and/or cognitive limitations [3]. In particular, people who have suffered stroke present deficits in balance. This is the main cause of the increased risk of falls and severe limitations suffered by patients in performing activities of daily living [3-5].

The deficit in balance experienced by patients who suffer stroke is due to loss of muscle strength and coordination and to spasticity and degenerative and neurological disorders [5]. The imbalance is visible in increased postural sway, in asymmetric distribution of weight between the legs at rest position, and in difficulty maintaining the center of mass in the limits of corporal stability during a task [1,3,6]. Due to their inability to recover from a loss of balance, patients who have suffered stroke have a high risk of falls [1,4,6]. Half of the people who have suffered stroke and are living in the community experience at least one fall per year, and about half of them suffer from repeated falls [1,4].

The Functional Reach Test (FRT) is a standardized instrument that assesses anteroposterior stability [7,8]. In recent years, it has been widely used to assess balance and risk of falls in people who have suffered a stroke [9]. It has proved to be an accurate, portable, cheap, and reliable test with low interexaminer variability [7,9,10].

Numerous studies have used inertial sensors as a tool for collecting kinematic data in the analysis of human motion in different functional tests, such as the Romberg test, the Time Up and Go test (TUG), the Sit to Stand test, and the FRT test [11-15]. Incorporating accelerometers and gyroscopes within the functions of mobile phones makes these devices the ideal replacement for inertial sensors as a tool for measuring human movement and imbalance through the instrumentalization of functional testing because of their portability, ease of use with apps, and low cost compared with inertial sensors [16-19]. Furthermore, in recent years, the mobile phone has emerged as an alternative to face-to-face health care for people living in different areas and with different pathologies, specifically in the diagnosis, assessment, intervention, and monitoring of patients (mHealth) [18,20-22].

There are no studies to date in which the FRT has been instrumentalized through a mobile device in people who have suffered a stroke. The aim of this study is to analyze the reliability of mobile phones for collecting kinematic variables in the parameterization of the FRT in people who have suffered a stroke. The hypothesis is that the mobile phone will be a reliable tool in the kinematic study of functional reach.

## Methods

### Design and Participants

This is an analytical cross-sectional study in which participants have suffered a stroke as defined by the World Health Organization [23]. The sample was selected considering the following inclusion criteria: age over 65 years of age, ability to walk for 10 meters at a speed equal to or higher than 0.8 m/s without help from another person or instrument support, capacity to stand upright without any help for 30 seconds, and moderate severity (score between 0 and 49 on Barthel’s Index). Exclusion criteria for this study were being 65 years of age, limitations in ambulation, major communication problems, severe cardiovascular, orthopedic or breathing limitations, having a secondary neurological disease, or failing to provide informed consent.

Ethical approval for the study was granted by the ethics committee of the Faculty of Health Sciences, University of Málaga. This study was conducted in accordance with Ethical Principles for Medical Research Involving Human Subjects (Helsinki Declaration 2008).

Before beginning the study, researchers gave each of the participants an information sheet and a request for informed consent, in which the study was explained, as well as the possibility that they may leave the study at any time, and an assurance of the protection of personal data, according to the Organic Law of Protection of Personal Data 19/55.

### Functional Reach Test

To perform the FRT or Duncan test (1990) [9], a tapeline is placed on the wall. The participant is then asked to situate themselves parallel to the tapeline, so that the axis through the participant’s shoulders is as perpendicular to the wall as possible. Their feet are located at the width of their shoulders, which are flexed 90º with elbows and hands outstretched. At this point, the researcher makes a mark on the tape using the metacarpal head of the third finger as a benchmark. From this starting position, the participant begins a movement for maximum anterior reach, before taking a step, lifting the heels, or touching the wall. A second mark on the wall is then made, and thereafter the participant returns to the starting position. The distance in centimeters between the two marks is the functional reach of each participant [7,9,10,24]. The reliability of this functional test is 0.81 [25].

In our study, a blinded investigator extracted the offline variables from each of the graphs generated after the collection of the kinematic data from each of the tests.

**Figure 1:**
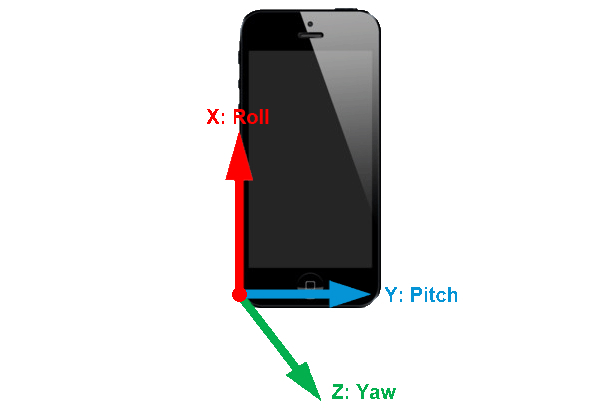
Origin of the coordinates (X, Y, Z) in the mobile.

During the execution of the FRT in this study, the participants each wore two mobile devices, one located at the L5–S1 (lumbar) level and the other at T7 (trunk). They were placed so that the origin of coordinates (X, Y, Z) (0, 0, 0) were placed in the left posterior-inferior vertex. See Figure 1.

### Mobile Devices

The two mobile devices used for the kinematic registration of the FRT were both iPhone 4s. This device has a triaxial gyroscope, accelerometer, and magnetometer [22,26,27]. The accelerometer was operated at a frequency of 32 Hz during the measurement. These accelerometers have a correlation coefficient of .98 or above [19,22]. We used SensorLog to retrieve sensor data for this study.

### Outcome Measures

The following variables were extracted from the FRT:

1. FRT distance: distance achieved by the participant between the starting position and the final position.
2. Maximum angular lumbosacral/thoracic displacement FRT: angular variation that the participant causes on the pitch axis. This amplitude is considered from the starting point until it reaches its peak before the return.
3. Time of maximum angular lumbosacral/thoracic displacement FRT: time it takes the participant to reach the peak.
4. Time for return to starting position: time it takes the participant to return to the starting point.
5. Total time FRT: time it takes the participant from the starting position to return to it.

These variables were taken from the kinematic registration of the mobile phone in the pitch axis.

Using data extracted previously, the following variables were calculated:

1. Average speed FRT: medium speed at which the test is run.
2. Maximum angular lumbosacral/thoracic displacement speed FRT: average speed at which the participant reaches the peak from the starting position.
3. Starting to return position speed: average rate at which the participant returns to the starting position from the peak.
4. Average acceleration FRT: average acceleration at which the participant executes the FRT.
5. Maximum angular lumbosacral/thoracic displacement average acceleration FRT: average acceleration at which the participant reaches the peak.
6. Acceleration average return starting position FRT: average acceleration the participant attained from the peak until the starting position.

The mean and the standard deviation of X, Y, Z were calculated in the maximum, minimum, and average speed and acceleration on both mobile devices. The result was found through the square root of the sum of the squares of the three axes in the displacement, the maximum and minimum speed, and the acceleration of the FRT, and also the mean and standard deviation in the result of the displacement and the result of the maximum and minimum speed and acceleration.

The variables analyzed were those we obtained from the repetition in which the participant achieved the widest functional reach.

### Procedure

At the beginning of the study, we explained to all participants what the test consisted of. Each signed the informed consent and completed the Barthel Index, the Stroke Impact Scale-16, and the Canadian neurological scale to improve the description of the sample. We also collected sociodemographic data on each of the participants via a questionnaire. The reliability of these tools are kappa=.93 [28], kappa=.76 [29], and intraclass correlation coefficient (ICC)=.70 to .92 [30], respectively.

**Figure 2:**
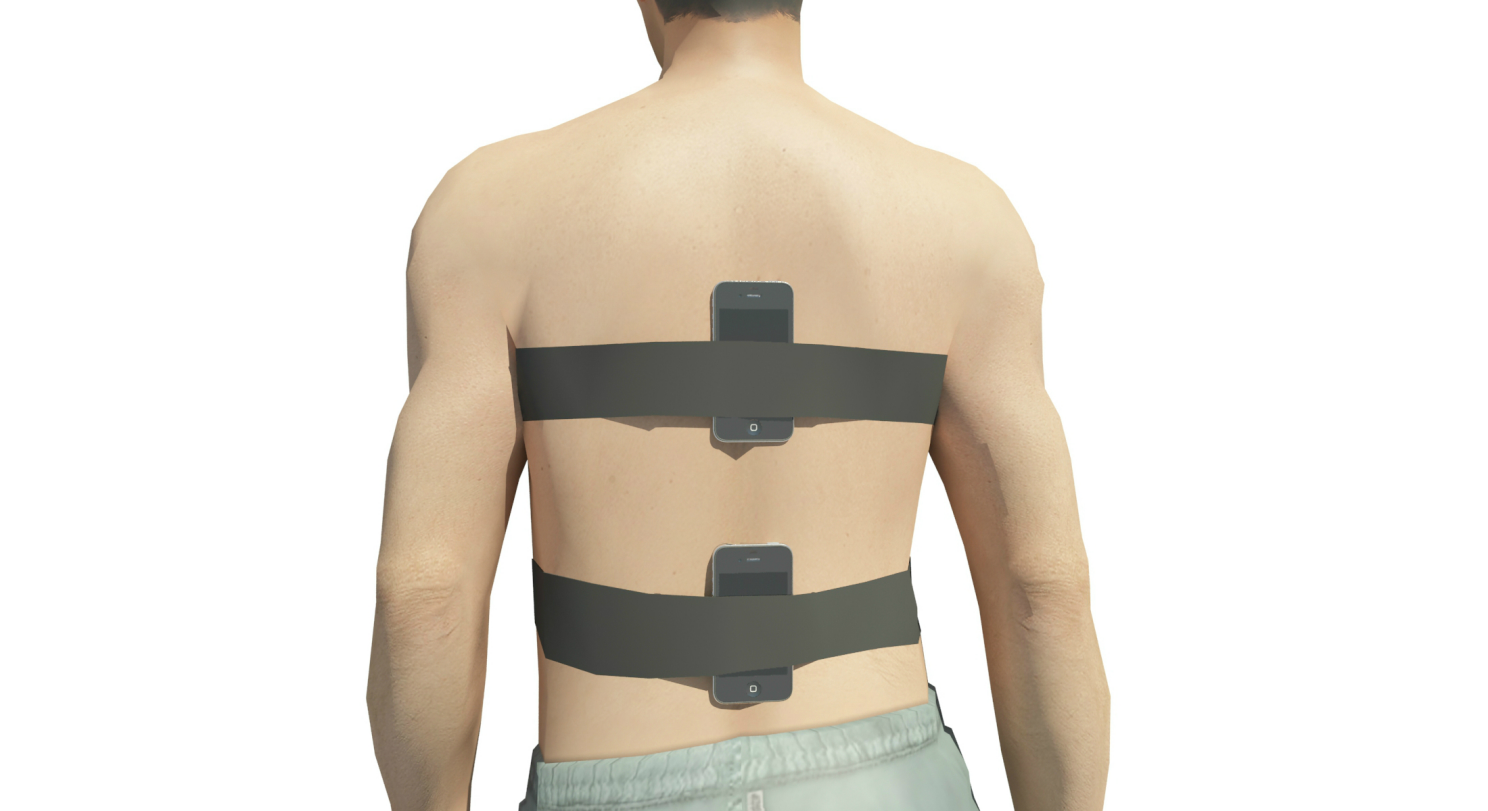
Position of the inertial sensors on the back of patients.

During the execution of the FRT or Duncan’s Test [9,25], the participants carried two mobile phones, one placed at the level of L5-S1 (lumbar) and the other at T7 (trunk). Three repetitions of the test were carried out under the supervision of 2 researchers. The 2 researchers then conducted the analysis of the results independently. See Figure 2.

From the kinematic registration collected by use of the mobile devices, we obtained the direct variables of time and displacement between the three intervals. As indirect variables, calculated thereafter, the velocity and displacement were obtained.

### Data Analysis

As noted above, sociodemographic data were collected through a questionnaire and a series of tests designed specifically for people with neurological disorders. Subsequently, the distance achieved in the FRT was recorded and a descriptive analysis of all kinematic variables recorded by both mobiles was conducted (trunk and lumbar).

The Kolmogov-Smirnov test was used to test the normality of the variables. The data obtained in the kinematic record from the trunk and lumbar positions were compared, both the direct variables (time and displacement) and the indirect variables (velocity, acceleration, and result). The Student *t* test was used for parametric variables and Wilcoxon’s test for nonparametric. The index of significance was set at *P*≤.05 values.

By analyzing internal consistency, we calculated the reliability of direct measurements with a confidence interval of 95% for each outcome variable. Correlation coefficients were calculated for interclass and intraclass reliability. Reliability was calculated for the reach achieved by the participant and direct variables measured by mobiles (time and displacement). The reliability of indirect variables (velocity, acceleration, and result) was not calculated because its value is determined by the reliability of direct measures. Levels of reliability were classified as follows: very low correlation was 0≤ICC≤.29, low correlation was .30≤ICC≤.49, moderate correlation was .50≤ICC≤.69, high correlation was .70≤ICC≤.89, and very high correlation was ICC≥.90 and above [31].

In this study, we used SPSS version 17.0 for Windows for statistical analysis.

## Results

Table 1 presents the demographic and anthropometric data collected through the questionnaire. It also shows the results of different specific tests used to obtain the degree of disability.

Table 2 shows the functional reach distance achieved by each participant and the description of the kinematic variables collected during the execution of the FRT depending on the position of the mobile, trunk, or lumbar. Furthermore, the registered movements appear divided into three intervals based on the start of the test, the maximum angular displacement, and the end of the test. This table shows the maximum, minimum, average and standard deviation of time, displacement, speed, and acceleration in each of the intervals.

**Table 1:** Descriptive values of participants*.*

	Minimum	Maximum	Mean	SD
Age in years	68	87	75.1	5.22
Canadian Neurological Scale	7.0	9.0	8.1	0.73
Barthel Index	80	100	93.50	5.95
Stroke Impact Scale-16	61	73	66.25	4.18
N valid (according to the list)	7			

**Table 2:** Description of the kinematic variables of FRT depending on the placement of the mobile device.^a^

		Minimum	Maximum	Mean	SD
Functional reach test distance in cm		9.86	16.84	13.15	2.49
**Trunk**					
	Time_A_B (s)	6.76	10.53	8.84	1.29
	Displacement_A_B (º)	6.24	18.90	12.62	5.19
	Speed_A_B (º/s)	0.49	2.49	1.43	0.79
	Acceleration_A_B (º/s^2^)	0.04	0.26	0.16	0.09
	Time_B_C (s)	4.73	10.55	7.18	2.74
	Displacement_B_C (º)	4.37	17.18	10.01	5.41
	Speed_B_C (º/s)	0.49	2.28	1.40	0.69
	Acceleration_B_C (º/s^2^)	0.08	0.22	0.19	0.11
	Time_A_C (s)	11.43	22.06	16.04	4.79
	Displacement_A_C (º)	14.01	31.82	22.64	7.87
	Speed_A_C (º/s)	0.62	2.16	1.36	0.72
	Acceleration_A_C (º/s^2^)	0.04	0.16	0.08	0.07
**Lumbar**					
	Time_A_B (s)	5.19	12.09	8.71	2.93
	Displacement_A_B (º)	6.40	16.02	10.93	4.02
	Speed_A_B (º/s)	0.76	1.48	1.25	1.07
	Acceleration_A_B (º/s^2^)	0.06	0.26	0.14	0.11
	Time_B_C (s)	4.24	11.58	7.81	3.16
	Displacement_B_C (º)	5.86	13.87	9.43	3.38
	Speed_B_C (º/s)	0.58	1.89	1.21	0.56
	Acceleration_B_C (º/s^2^)	0.06	0.26	0.15	0.08
	Time_A_C (s)	10.48	22.97	16.52	5.11
	Displacement_A_C (º)	11.59	28.20	20.36	7.20
	Speed_A_C (º/s)	0.72	1.68	1.24	1.04
	Acceleration_A_C (º/s^2^)	0.03	0.12	0.07	0.04
N valid (according to the list)		7			

^a^A: beginning of the FRT; B: maximum angular displacement; C: end of the FRT.

Table 3 shows the result of the displacement, of the maximum and minimum speed and acceleration in the FRT; and the average, maximum, and minimum speed and acceleration. The variables were presented as the mean and standard deviation of the sum of the participants in relation to the three axes of each mobile and the difference between them.

Table 4 presents the intraobserver and interobserver reliability with a 95% confidence interval for each of the direct variables obtained in the instrumentalization of the FRT by mobile. They are presented according to the placement of the mobile device and divided into three intervals of movement.

**Table 3:** Mean and standard deviation of the records of each of the sensors and differences between them.

	Trunk (SD)			Lumbar (SD)			Mean difference (SD)		
	X	Y	Z	X	Y	Z	X	Y	Z
Resultant displacement	34.92 (7.02)			37.06 (14.75)			1.86^a^ (23.64)		
Speed mean	1.79 (0.27)	25.44 (7.84)	24.59 (8.73)	1.68 (0.67)	22.39 (7.42)	19.42 (5.03)	0.11^a^ (0.74)	3.05^a^ (1.97)	5.17^a^ (8.43)
Speed maximum	-0.57 (0.70)	10.06 (3.97)	9.38 (1.42)	1.48 (0.94)	9.76 (6.14)	8.11 (1.07)	-2.05^a^ (0.62)	0.30^b^ (3.51)	-1.27^b^ (1.74)
Speed minimum	-2.19 (0.73)	-15.79 (2.81)	-13.10 (7.49)	1.19 (1.16)	-14.18 (4.43)	-12.28 (3.86)	-3.38^a^ (1.19)	1.61^b^ (4,07)	-0.82^b^ (9.21)
Resultant speed maximum	13.80 (4.22)			13.19 (4.70)			-0.61ª (4.41)		
Resultant speed minimum	20.55 (5.61)			19.01 (4.18)			-1.54^a^ (2.74)		
Aceleration mean	2.34 (1.21)	3.03 (1.27)	6.53 (1.32)	1.39 (1.01)	0.43 (3.38)	5.27 (1.84)	0.95^a^ (0.98)^c^	2.60^b^ (3.83)	1.26^b^ (1.96)
Aceleration maximum	0.73 (0.81)	2.19 (3.07)	95.40 (8.54)	0.43 (0.29)	2.34 (2.13)	90.94 (5.09)	0.30^a^ (1.43)	-0.15^b^ (3.79)	4.46^a^ (6.05)
Aceleration minimum	-2.42 (2.26)	4.33 (2.72)	88.88 (9.58)	1.84 (1.17)	2.97 (3.07)	84.11 (7.07)	-4.26^b^ 3.18)	1.36^a^ (2.81)^d^	4.77^b^ (6.18)
Resultant acceleration maximum	88.17 (10.23)			89.51 (8.69)			-1.34^b^ (4.84)		
Resultant acceleration minimum	90.19 (9.28)			88.71 (7.91)			1.48^a^ (5.71)		

^a^Differences calculated through Student *t* test (parametric distribution of the sample).

^b^Differences calculated through Wilcoxon’s test (nonparametric distribution of the sample).

^c^ *P*=.02.

^d^ *P*=.03.

**Table 4:** Intraobserver and interobserver reliability of variables measured directly during FRT.

Variable			Intraobserver		Interobserver	
			ICC	95% CI	ICC	95% CI
**Trunk**						
	**Time**					
		A_B	.872	.857-.886	.868	.857-.875
		B_C	.847	.831-.862	.840	.835-.851
		A_C	.884	.873-.892	.864	.853-.876
	**Displacement**					
		A_B	.884	.871-.894	.873	.867-.880
		B_C	.870	.862-.879	.861	.854-.872
		A_C	.880	.869-.887	.869	.857-.882
**Lumbar**						
	**Time**					
		A_B	.883	.874-.891	.871	.864-.878
		B_C	.867	.855-.876	.853	.848-.860
		A_C	.849	.833-.860	.842	.837-.849
	**Displacement**					
		A_B	.874	.862-.887	.861	.853-.869
		B_C	.877	.864-.885	.864	.857-.873
		A_C	.869	.859-.883	.857	.850-.864
Functional Reach Test			.989	.981-.996	.978	.970-.985

## Discussion

### Principal Findings

The results show that inertial sensor mobile phones can be an accurate and reliable instrument for obtaining kinematic variables in the instrumentalization of FRT in people who have suffered a stroke.

The reliability of this study can be classed as high correlation [31], with ranges in intraobserver reliability between .831 and .894 and interobserver reliability between .835 and .882 (Table 4). These values are shown to be in accordance with values observed in previous and similar studies. Marchetti et al [12] had test-retest reliability of .87 (.68-.95), Merchán-Baeza et al [15] showed intraobserver reliability of .829-.878 and interobserver of .821-.883, and Mellone et al [14] had intraobserver reliability of .72 (.46-.86) and interobserver of .99 (.99-1.00). In the latter study, the reliability was extracted during the execution of a specific section of the TUG test, namely, from sitting to standing [14]. The differences in reliability between the values of our study and that of Mellone et al [14] could be due to the type of balance analyzed in each test. In our study, the controlled semistatic equilibrium was analyzed, whereas Mellone et al [14] analyzed the coordinated and explosive semistatic equilibrium necessary for carrying out a normal gesture [14]. However, the interobserver reliability cannot be compared between this study and that of Mallone et al because they did not differentiate in the calculation of reliability distinct values for the mobile device and for the accelerometer [14].

The high reliability observed in the duration of our test (ie, intraobserver reliability of .847-.884 and interobserver of .840-.871) is comparable with the results shown by Mellone et al [14] in the parameterization of the TUG with a mobile device, with an ICC value of .83-.96 for intraobserver and 1.00-1.00 for interobserver. Although in the latter study the value for the accelerometer and mobile device was unified. Merchán-Baeza et al [15] had ICC values of .806-.880 (intraobserver) and .804-.879 (interobserver).

Given the position where the mobile phone is located in our study, the values of intraobserver reliability ranged between .847 and .884 for the trunk and between .849 and .883 for the lumbar position data. These were in accordance with the results obtained from a previous study where there were no observed notable differences in the values of reliability when two inertial sensors were placed in the same segments as our study (trunk and lumbar) for the kinematic record of the FRT. The ICC values observed in that study [15] were .835-.877 (trunk) and .829-.878 (lumbar). In addition, the mobile data are stable not only in primary measures, but also in secondary measures, as shown by Nishiguchi et al [13]: peak frequency ICC=.906, 95% CI .83-.95; root mean square ICC=.902, 95% CI .82-.95; autocorrelation peak ICC=.752, 95% CI .55-.87, and coefficient of variance ICC=.777, 95% CI .59-.89.

### Strengths and Limitations

The main weakness in this study is the sample size, which is small, but sufficient to provide evidence for usefulness of mobile devices in the kinematic record of FRT in people who have suffered stroke. However, it would be beneficial to increase the number of participants to consolidate the results. Future studies should make absolute comparisons between healthy people and people with a profile marked by a static, semistatic, or dynamic imbalance during the FRT. However, a particular strength of our study is that it is the first to perform simultaneous kinematic recording using two mobile devices, that is, one placed on the trunk and another in the lumbar position.

### Conclusions

Mobile phones have been proven to be reliable, valid, and specific tools to analyze the kinematics in FRT parameterization. Besides these properties, it is important to also note economy, ease of access, ease of use, portability, no computer needed to record the registration, large internal memory, stored data can be sent by email instantaneously, and additionally there are numerous apps to optimize the use of the various elements of the device. For these reasons, it can be argued that mobile devices have greater clinical potential than the inertial sensors (or accelerometers) commonly used in the laboratory. These statements supplement other similar claims made in previous studies [13,14].

We conclude that mobile phones are reliable tools for parameterization of FRT in people who have suffered a stroke.

## Abbreviations

FRT: Functional Reach Test
ICC: intraclass correlation coefficient
TUG: Time Up and Go test
